# Supplementary material for: PlrA (MSMEG_5223) is an essential polar growth regulator in Mycobacterium smegmatis
Source: PLoS One. 2023 Jan 12;18(1):e0280336. doi: 10.1371/journal.pone.0280336 (PMC9836265; doi:10.1371/journal.pone.0280336)
Supplement: S1 File — (DOCX) [file pone.0280336.s001.docx]

Supplemental Table 1. Strains.

| **Strains** | | | |
| --- | --- | --- | --- |
| Strain # | nickname | genotype | Figure panel |
| CB966 | Ptet:: *plrA* | mc^2^155 ∆ *plrA*::zeoR L5::pMC1s- *plrA* | 1 |
| CB913 | *plrA* -GFPmut3 | mc^2^155 L5::pCT94- *plrA* -GFPmut3 | 2 |
| CB2642 | ∆*plrA* L5::*plrA*-strep | mc^2^155zeoR::∆*plrA* L5::pCT94-*plrA*-strep | 3BCDEF |
| CB2645 | ∆plrA L5:: *plrA∆*CT-strep | mc^2^155zeoR::∆ *plrA* L5::pCT94- *plrA* ∆CT-strep | 3BCDEF |
| CB2656 | mc^2^155 L5::*plrA*-strep | mc^2^155 L5:: pCT94-*plrA* -strep | 3C |
| CB2657 | mc^2^155 L5::*plrA*∆CT-strep | mc^2^155 L5:: pCT94 - *plrA*∆CT-strep | 3C |
| CB2658 | mc^2^155 L5::*plrA*∆NT-strep | mc^2^155 L5:: pCT94- *plrA* ∆NT-strep | 3C |
| CB2660 | Ptet:: *plrA* Wag31-RFP | mc2155 zeoR::∆ *plrA* L5::pCT16- *plrA* / pMEK-Ptb21-Wag31-RFP | 4 |

Supplemental Table 2. Plasmids.

*If a published vector was used unaltered, it is indicated with an * in the “Ref for parent/vector” column.

| **Plasmids.** | | | | |
| --- | --- | --- | --- | --- |
| strain # | Plasmid name | Description of plasmid | Used in strains | Ref for parent/ vector* |
| CB964 | pMC1s- *plrA* | L5 integrating, nuoR, ptetO promoter driving expression of *plrA*, expresses the tetR repressor constitutively. | CB966, CB2321 | [1] |
| CB909 | pCT94- *plrA*-GFPmut3 | L5 integrating, kanR, ptetO promoter driving expression of *plrA-*GFPmut3 | CB1126 | [2] |
| CB1401 | pCT94-MSMEG_5223-strep | L5 integrating, kanR, ptetO promoter driving expression of *plrA-*strep | CB2656, CB2642 | [2] |
| CB2636 | pCT94 - 5223- ∆ CT-strep | L5 integrating, kanR, ptetO promoter driving expression of *plrA*∆CT*-*strep (only residues 1-117) | CB2657, CB2645 | [2] |
| CB2637 | pCT94 - 5223- ∆ NT-strep | L5 integrating, kanR, ptetO promoter driving expression of *plrA*∆NT*-*strep (only residues 118-368) | CB2658 | [2] |
| CB 1261 | pMEK-Ptb21-Wag31-mRFP | Episomal, kanR, Ptb21 promoter driving expression of Wag31-mRFP | CB2660 | [3]* |

| **Primers** | | |
| --- | --- | --- |
| Strain # | Feature | primers |
| CB966 | ∆ *plrA*::zeoR | GGCCAGTGAATTACTTAAGAGATCTtcgtcgtcgttgaagacc |
|  |  | ATAGCATACATTATACGAAGTTATacgtagcagaagccgaagac |
|  |  | gtcttcggcttctgctacgtATAACTTCGTATAATGTATGCTAT |
|  |  | cggtactccgaacgatgatcATAACTTCGTATAGCATACATTATA |
|  |  | TATAATGTATGCTATACGAAGTTATgatcatcgttcggagtaccg |
|  |  | CTATGACCATGATTACGCCAAGCTTctctcacagaccacgctgag |
|  | pMC1s- *plrA* | CTTAATTAAGAAGGAGATATATCGATgccgtggtggggtgccgtgttg |
|  |  | AGATATCCATGGATCCAGCTGCAGAATtcagtcccgcgagtgacggcc |
| CB913 | pCT94- *plrA* -GFPmut3 | AATGAGCACGATCCGCATGCTTAATTAAGAAGGAGGATATCatg  ccgtggtggggtgccgt |
|  |  | CAGTGAAAAGTTCTTCTCCTTTACTGGTACCgtcccgcgagtgacggcc  ccgc |
|  |  | gggccgtcactcgcgggacGGTACCAGTAAAGGAGAAGAACTTTTCAC |
|  |  | GGTCCCCAATTAATTAGCTAAAGCTTtcaTTTGTATAGTTCAT  CCATGCCATGT |
| CB2656 | pCT94-*plrA*-strep | GCATGCTTAATTAAGAAGGAGATATACATatgccgtggtggggtgccgtgttggcggct |
|  |  | AACTGGGGGTGGCTCCAGTCGGCGCCGGTGGAGTGGATATCgtcccgcgagtgacggcc |
|  |  | TAGGGTCCCCAATTAATTAGCTAAAGCTTTCACTTCTCGAACTGGGGGTGGCTCCAGTC |
| CB2657 | pCT94 – *plrA* ∆CT-strep | GCATGCTTAATTAAGAAGGAGATATACATatgccgtggtggggtgccgtgttggc |
|  |  | CTAGGGTCCCCAATTAATTAGCTAAAGCTTCTACTTCTCGAACTGGGGGTGGCTCCAataccagcggcccagcccgatcagc |
| CB2658 | pCT94- *plrA*∆NT-strep | GCATGCTTAATTAAGAAGGAGATATACATatggtcgggctgacgtcccgcgacaccg |
|  |  | AACTGGGGGTGGCTCCAGTCGGCGCCGGTGGAGTGGATATCgtcccgcgagtgacggcc |
|  |  |  |

References:

1. Wei J-R, Krishnamoorthy V, Murphy K, Kim J-H, Schnappinger D, Alber T, et al. Depletion of antibiotic targets has widely varying effects on growth. PNAS. 2011;108: 4176–4181. doi:10.1073/pnas.1018301108

2. Kieser KJ, Boutte CC, Kester JC, Baer CE, Barczak AK, Meniche X, et al. Phosphorylation of the Peptidoglycan Synthase PonA1 Governs the Rate of Polar Elongation in Mycobacteria. Behr MA, editor. PLOS Pathogens. 2015;11: e1005010. doi:10.1371/journal.ppat.1005010

3. Meniche X, Otten R, Siegrist MS, Baer CE, Murphy KC, Bertozzi CR, et al. Subpolar addition of new cell wall is directed by DivIVA in mycobacteria. Proceedings of the National Academy of Sciences. 2014;111: E3243–E3251. doi:10.1073/pnas.1402158111
