# Supplementary figures and images for: PlrA (MSMEG_5223) is an essential polar growth regulator in Mycobacterium smegmatis
Source: PLoS One. 2023 Jan 12;18(1):e0280336. doi: 10.1371/journal.pone.0280336 (PMC9836265; doi:10.1371/journal.pone.0280336)

Original image file of western blot cropped to make Fig. 3C

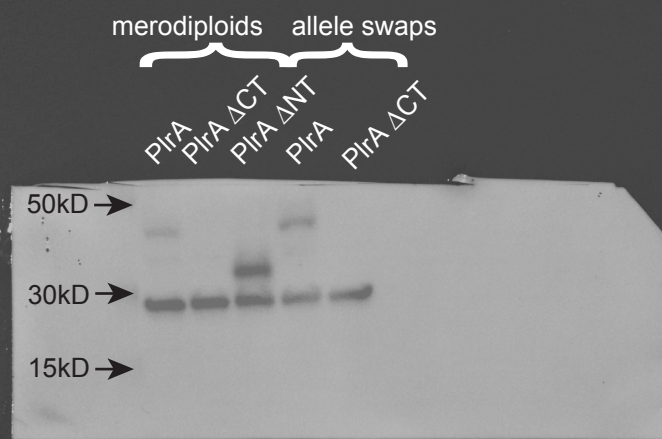

Supplement: S1 Raw image — (PDF) [file pone.0280336.s002.pdf]
